# Evaluation of monocyte to high-density lipoprotein ratio, lymphocytes, monocytes, and platelets in psoriasis^☆^^☆☆^

**DOI:** 10.1016/j.abd.2019.05.002

**Published:** 2019-12-09

**Authors:** Ezgi Aktaş Karabay, Damla Demir, Aslı Aksu Çerman

**Affiliations:** a Department of Dermatology and Venereology, Faculty of Medicine, Bahçeşehir University, İstanbul, Turkey; b Department of Dermatology and Venereology, Sisli Hamidiye Etfal Training and Research Hospital, Health Sciences University, Istanbul, Turkey

**Keywords:** High-density lipoprotein C, Lymphocyte, Monocyte, Platelet, Psoriasis

## Abstract

**Background:**

Psoriasis is a chronic immune-mediated inflammatory skin disease that is associated with cardiovascular comorbidities.

**Objectives:**

The objective of this retrospective study is to assess the C-reactive protein, monocyte-to-high-density-lipoprotein ratio, neutrophil-to-lymphocyte ratio, platelet-to-lymphocyte ratio, and monocyte-to-lymphocyte ratio as inflammatory markers in patients with psoriasis and to search for a relationship between these parameters and psoriasis severity, as defined by the psoriasis area and severity index.

**Methods:**

There were 94 patients with psoriasis and 118 healthy controls enrolled in the study. The C-reactive protein, monocyte-to-high-density-lipoprotein ratio, neutrophil-to-lymphocyte ratio, platelet-to-lymphocyte ratio, and monocyte-to-lymphocyte ratio values of two groups were retrospectively evaluated.

**Results:**

Statistically significant differences were observed in terms of C-reactive protein, monocyte-to-high-density-lipoprotein ratio, neutrophil-to-lymphocyte ratio and monocyte-to-lymphocyte ratio between the patient and control groups (p = 0.001, p = 0.003, p = 0.038, and p = 0.007, respectively). Positive correlations were found between the psoriasis area and severity index and the values of C-reactive protein, monocyte-to-high-density-lipoprotein ratio, neutrophil-to-lymphocyte ratio, platelet-to-lymphocyte ratio, and monocyte-to-lymphocyte ratio (r: 0.381; p < 0.01, r: 0.203; p < 0.05, r: 0.268; p < 0.01, r: 0.374; p < 0.01, r: 0.294; p < 0.01, respectively).

**Study limitations:**

The small sample size and the retrospective design of the study are limitations.

**Conclusion:**

Elevated C-reactive protein, monocyte-to-high-density-lipoprotein ratio, neutrophil-to-lymphocyte ratio, and monocyte-to-lymphocyte ratio were significantly associated with psoriasis. A positive correlation between C-reactive protein and monocyte-to-high-density-lipoprotein ratio leads to the suggestion that monocyte-to-high-density-lipoprotein ratio might be a reliable parameter in psoriasis during the follow-up. The relationship between the diasease and inflammatory parameters might provide early detection of cardiovascular morbidities in psoriasis patients.

## Introduction

Psoriasis, a chronic skin disease, is characterized by the infiltration of immune cells (*e.g*., mononuclear leukocytes and neutrophils) into the skin, as well as by abnormal proliferation of keratinocytes.^1^ It is well known that psoriasis is associated with an increased risk of cardiovascular diseases (CVD), as they share common inflammatory pathways.^2^

Levels of C-reactive protein (CRP), which is a well-known biomarker of inflammation, have been shown to be elevated in patients with psoriasis, indicating cardiovascular comorbidities.^3^ The ratio of the monocyte count to high-density lipoprotein cholesterol (MHR) has been demonstrated to be an inflammatory marker, and a new predictor and prognostic factor for CVDs.^4^ Moreover, the neutrophil–lymphocyte ratio (NLR), platelet-lymphocyte ratio (PLR), and monocyte–lymphocyte ratio (MLR) have been identified as indicators of systemic inflammation and poor prognosis in a variety of diseases, such as cancer, CVD, and autoimmune inflammatory diseases.^5^, ^6^, ^7^ However, these novel biomarkers have not been well investigated in psoriasis.

Several biomarkers have been studied in psoriatic patients’ peripheral blood samples to find an adequate serum marker to evaluate the severity of psoriasis. The Psoriasis Area and Severity Index (PASI) is a scoring tool that is used to assess the severity of psoriasis,^8^ but it may show variations according to the physician. Therefore, it would be beneficial to find an objective, inexpensive, and easily accessible clinical biomarker in psoriasis. However, no well-accepted biomarkers have been established for psoriasis.

To the best of the authors’ knowledge, no current data is available on MHR or MLR values in patients with psoriasis, and only a few studies have investigated NLR and PLR values in psoriasis.^6^, ^9^, ^10^, ^11^, ^12^ Therefore, to better understand these serum inflammatory parameters in psoriasis and to evaluate the association with the disease activity, a retrospective study to assess CRP, MHR, NLR, PLR, and MLR all together in psoriasis was conducted.

## Methods

Ninety-four patients diagnosed with chronic plaque psoriasis by clinical or histopathological examination were recruited from the dermatology outpatient clinic. For comparison, 118 healthy age- and gender-matched controls with no evidence of psoriasis or other systemic inflammatory disease were recruited from among hospital staff volunteers. Subjects with a history of any topical or systemic psoriasis therapy were excluded from the study. Only those with normal body mass index (BMI) (18.5–25 kg/m^2^) were included. The control subjects had normal findings on physical examination, and none had CVD or disease in any other organ systems. Subjects were excluded if they had a documented history of any other systemic disease, including coronary artery disease, myocardial infarction, hypertension, hyperlipidemia, stroke, peripheral vascular disease, chronic obstructive pulmonary disease, renal disease, liver failure, or clinical/biochemical evidence of concomitant inflammatory disease or any autoimmune disease. Subjects under any treatment were excluded. Subjects with smoking and/or alcohol consumption habits were also excluded.

The study was reviewed and approved by the local ethics committee, and all the individuals provided written informed consent. The study was carried out according to the principles expressed in the Declaration of Helsinki.

Data on the baseline demographics, clinical characteristics, and blood test results were obtained retrospectively from the patient charts. Levels of high-density lipoprotein cholesterol (HDL-C), complete blood count (CBC), and CRP were measured in all subjects using fasting venous blood samples. Venous blood samples were drawn from the participants between the hours of 9:00 and 11:00 a.m. following a 12-h fasting period to assess CBC, HDL-C, and CRP. The MHR, NLR, PLR, and MLR levels of the subjects were also recorded.

Measurements of serum CRP levels were performed with a spectrophotometric system (Cobas c 501; Roche Diagnostics – Mannheim, Germany). Hemogram tests were assessed by an auto-analyzer system (Sysmex XE-2100; Roche Diagnostics – Mannheim, Germany). HDL concentrations were measured with an enzymatic system (Cobas c 701; Roche Diagnostics – Mannheim, Germany).

Psoriasis was graded according to the PASI, calculated at the time of blood collection by a single dermatologist.^8^ The psoriasis patients were divided into two groups according to PASI scores: mild (≤10) and moderate–severe (>10). The MHR, NLR, PLR, MLR, and CRP levels of psoriasis patients were compared with both controls and intragroup comparisons were made according to the severity of the disease.

### Statistical analysis

The Number Cruncher Statistical System 2007 program (NCSS; Kaysville – Utah, United States) was used for the statistical analysis. The data were expressed with mean ± standard deviation, median (first quartile, third quartile), count, and percentages. In the analysis of normally distributed variables, Student's *t*-test for independent samples was applied to examine the differences between two groups. The differences between two independent groups were examined using the Mann–Whitney *U* test for non-normally distributed variables. One-way ANOVA, with Bonferroni corrected *post hoc* tests, was used to compare normally distributed variables between three or more groups. The Kruskal–Wallis test, with Dunn-Bonferroni *post hoc* tests, was used to compare non-normally distributed variables between three or more groups. Pearson's chi-squared test was used to compare categorical variables. The association between variables was evaluated by Spearman's rank correlation coefficient. A *p*-value of < 0.05 was considered statistically significant.

## Results

A total of 94 patients with psoriasis and 118 healthy control subjects were included in the study. The psoriasis group consisted of 50 patients with mild disease and 44 patients with moderate–severe psoriasis. No significant differences were observed in the gender ratio or age between the patients with psoriasis and healthy controls (*p* = 0.393, *p* = 0.222, respectively). All the participants were in the normal limits of BMI. The median (first quartile, third quartile) value for disease duration was 120 (72–180) months among the psoriasis patients. The PASI scores of the patients ranged from 0.6 to 32.4, with a median (first quartile, third quartile) value of 9.65 (4.9–15.4).

A statistically significant difference in CRP levels was noted between the patient and control groups: 3.3 (3–5) *vs.* 3 (1.37–3.44), *p* = 0.001, respectively. A statistically significant difference was also observed in terms of MHR, NLR, and MLR between the patient and control groups (*p* = 0.003, *p* = 0.038, and *p* = 0.007, respectively). No statistically significant difference was shown in the values of PLR between the two groups (*p* = 0.865). The clinical characteristics of the study group are shown in Table 1

**Table 1:** Demographic features and values of inflammatory parameters of the patients and controls.

	Psoriasis (*n* = 94)	Controls (*n* = 118)	*p*
Age (years)	38.28 ± 12.48	36.21 ± 11.98	0.222
CRP	3.3 (3–5)	3 (1.37–3.44)	0.001
MHR	0.011 (0.007, 0.016)	0.009 (0.006–0.012)	0.003
NLR	1.96 (1.65, 2.34)	1.77 (1.31–2.43)	0.038
PLR	106.94 (90–141.91)	110.79 (90.61–141.5)	0.865
MLR	0.23 (0.18–0.29)	0.2 (0.15–0.25)	0.007

Student's *t*-test for independent samples, reported as mean ± SD.

Mann–Whitney *U* test, reported as median (first quartile, third quartile).

CRP, C-reactive protein; MHR, monocyte-to-high-density-lipoprotein ratio; NLR, neutrophil-to-lymphocyte ratio; PLR, platelet-to-lymphocyte ratio; MLR, monocyte-to-lymphocyte ratio.

When patients with psoriasis were evaluated according to disease severity, the values of CRP, MHR, NLR, PLR, and MLR were significantly higher in patients with moderate–severe psoriasis than in patients with mild psoriasis and healthy controls (*p* = 0.001, *p* = 0.003, *p* = 0.024, *p* = 0.019, and *p* = 0.002, respectively) (Table 2).

**Table 2:** Inflammatory parameters according to the severity of psoriasis.

	Mild psoriasis (PASI ≤ 10) (*n* = 50)	Moderate–severe psoriasis (PASI > 10) (*n* = 44)	Controls (*n* = 118)	*p*
Age (years)	38.28 ± 12.48	37.36 ± 10.99	36.21 ± 11.98	0.378
CRP	3 (2.87–4.17)^a^	3.5 (3.3–5)^b^	3 (1.37–3.44)^a^	0.001
MHR	0.01 (0.01–0.01)^a^	0.01 (0.01–0.02)^b^	0.01 (0.01–0.01)^a^	0.003
NLR	1.94 (1.59–2.18)	2.02 (1.69–2.86)^b^	1.77 (1.31–2.43)^a^	0.024
PLR	101.71 (85.15–119.06)^a^	125.22 (99.34–159.6)^b^	110.79 (90.61–141.5)	0.019
MLR	0.22 (0.17–0.26)	0.27 (0.19–0.35)^b^	0.2 (0.15–0.25)^a^	0.002

a One-way ANOVA with Bonferroni corrected *post hoc* tests, reported as mean ± SD.

b Kruskal–Wallis test with Dunn-Bonferroni *post hoc* tests, reported as median (first quartile, third quartile). ^a,b^ Equal letters do not differ by the Dunn test at 5% significance. CRP, C-reactive protein; MHR, monocyte-to-high-density-lipoprotein ratio; NLR, neutrophil-to-lymphocyte ratio; PLR, platelet-to-lymphocyte ratio; MLR, monocyte-to-lymphocyte ratio.

A positive correlation was seen between PASI score and the values of CRP, MHR, NLR, PLR, and MLR (*r* = 0.381, *p* = 0.001; *r* = 0.203, *p* = 0.045; *r* = 0.268, *p* = 0.009; *r* = 0.374, *p* = 0.001; *r* = 0.294, *p* = 0.004, respectively).

A positive correlation was noted between the values of CRP and MHR (*r* = 0.273, *p* = 0.001). MHR and NLR values were positively correlated (*r* = 0.163, *p* = 0.018), while a negative correlation was noted between the values of MHR and PLR (*r* = −0.161, *p* = 0.019). A positive correlation was observed between the values of NLR and PLR (*r* = 0.461, *p* = 0.001). No other statistically significant relation was noticed between the parameters (between CRP and NLR, *p* = 0.243; CRP and PLR, *p* = 0.116).

No correlations were found between the values of CRP, MHR, NLR, PLR, and MLR and disease duration (*p* = 0.164, *p* = 0.056, *p* = 0.190, *p* = 0.584, *p* = 0.091, respectively).

## Discussion

This study demonstrated elevated levels of CRP, MHR, NLR, and MLR, and a positive correlation between the disease severity and the values of CRP, MHR, NLR, PLR, and MLR. Interestingly, CRP, a well-known biomarker of inflammation, was only correlated with MHR. Psoriasis is a chronic immune-mediated inflammatory skin disease associated with cardiovascular comorbidities. Many inflammatory cells and cytokines are involved in the pathogenesis of the disease.^1^, ^2^ Tumor necrosis factor (TNF)-α, interferon (IFN)-α, IFN-g, interleukin (IL)-1β, IL-6, IL-12, IL-17A, IL-17F, IL-22, and IL-23 from various immune cells, primarily T helper type 1 (Th1) and type 17 (Th17) cells, also contribute to the pathophysiology of psoriasis, as well as keratinocytes, other T cells, and dendritic cells.^13^ Histological studies of psoriatic lesions have revealed infiltration of T lymphocytes and neutrophils in psoriatic lesions.^6^ Additionally, some reports have shown increased neutrophil, monocyte, and lymphocyte counts in the peripheral blood of patients with psoriasis.^13^ Platelets have also been shown to play a role in immunological inflammatory reactions.^14^

Recently, studies have revealed the association between psoriasis and indicators of subclinical CVD, such as higher carotid intima media thickness, greater coronary artery calcification, higher arterial stiffness, and endothelial dysfunction^15^; in addition, improvement in psoriasis severity has been suggested to be related with reductions in vascular inflammation.^16^ Atherosclerosis, the pathological process underlying CVD and psoriasis, shares common pathogenic features, including local and systemic immunological processes, inflammatory cytokine/chemokine profiles, and inflammatory markers.^2^ The main causes of morbidity and mortality of patients with psoriasis were reported to be cardiovascular events,^15^ which makes it even more desirable to find an appropriate tool to define cardiovascular risks in psoriasis.

C-reactive protein (CRP), an acute phase protein, is produced under the influence of cytokines such as IL-6 and TNF-α. Elevated CRP concentration was reported to be related to increased risk of CVD.^17^ In several studies, a higher level of CRP has been demonstrated in psoriasis as an inflammatory condition.^3^ Coimbra et al.^18^ showed a positive correlation between CRP and PASI, and suggested that elevated CRP levels may be a predictor of future CVDs. Other studies have not found a significant correlation between CRP and PASI scores.^19^ In the present study, CRP levels were also higher in patients with psoriasis and were found to be associated with the PASI score.

Psoriasis patients may present CVD risk factors, including decreased levels of HDL-C, which may be related with the severity of psoriasis.^20^ HDL-C has been shown to suppress the pro-oxidant and proinflammatory effects of monocytes. HDL-C also inhibits the proliferation and differentiation of progenitor cells of monocytes, resulting in a reduction in monocyte activity.^21^ Monocytes play a major role in the progression of atherosclerosis *via* interaction with activated endothelium, which results in the overexpression of inflammatory cytokines, including the monocyte chemotactic protein 1 ligand, vascular cell adhesion molecule 1, and intercellular adhesion molecule 1. Subsequently, macrophages that are differentiated from monocytes ingest oxidized LDL-C, and the initial foam cells occur.^22^ However, HDL-C molecules remove cholesterol debris from macrophages. Through the interactions with monocytes, HDL-C molecules show anti-inflammatory and antioxidant effects.^23^ Therefore, increased monocyte and decreased HDL-C have been suggested to be related with inflammation. Recently, MHR has been demonstrated to be an inflammatory marker and a new predictor and prognostic factor for CVD.^4^, ^21^ Kanbay et al.^24^ demonstrated higher MHR to be associated with poor cardiovascular prognosis in renal disease, and MHR was reported to be a predictor of atrial fibrillation after catheter ablation in another study.^6^ Açıkgöz et al.^23^ reported MHR to be associated with endothelial dysfunction in Behçet's disease (BD) and suggested MHR as a marker of inflammation as well as an early predictor of vascular involvement in patients with BD. Some studies have reported higher HDL-C values in psoriasis patients.^25^ However, no studies to date have investigated MHR in patients with psoriasis. The present study found higher MHR in patients with psoriasis compared with the controls, and a positive correlation was observed between MHR and the PASI score. Moreover, MHR and CRP levels were positively correlated, which leads to the suggestion that MHR might be a reliable marker of systemic inflammation in psoriasis.

NLR, PLR, and MLR have been identified as indicators of systemic inflammation and poor prognosis in a variety of diseases, such as cancer, CVD, and autoimmune inflammatory diseases.^5^, ^6^, ^7^ These ratios are more stable than the individual blood cell counts and are less affected by conditions that change the individual cell counts.^6^

Previously, NLR, PLR, and MLR have been studied in diabetes mellitus, acute coronary syndrome, ulcerative colitis, end-stage renal disease, tuberculosis, rheumatoid arthritis, cirrhosis, systemic inflammation, familial Mediterranean fever, and in some cancers to determine prognoses.^7^, ^26^, ^27^, ^28^

A few studies have evaluated NLR and PLR in psoriasis patients, with varying results. Previously increased NLR levels in psoriasis patients has been reported,^9^, ^10^, ^11^, ^12^ and some reports have shown a positive correlation between PASI and NLR,^9^, ^10^ which is consistent with the present findings. However, in another study conducted by Ataseven et al.,^11^ no association between NLR and PASI score was demonstrated. In the present study, NLR was found to be elevated in psoriasis and associated with PASI scores.

An association between increased platelet activation and atherosclerosis has been reported^14^ and some studies have shown a significant elevation of PLR in patients with psoriasis.^12^ Kim et al.^9^ showed a significant relationship between PASI score and PLR, while the values of PLR showed no differences between psoriasis patients and controls. Similarly, the present study did not find any significant difference in PLR values between psoriasis patients and controls, but a positive correlation was observed between PLR and PASI scores.

Currently, MLR has been demonstrated as an indicator of systemic inflammation and a marker of disease severity in many diseases, such as BD, cancer, and axial spondyloarthritis.^29^, ^30^ In the literature, no studies evaluating MLR in psoriasis were noted, but the present study found higher MLR levels in patients with psoriasis than controls and a positive correlation between MLR and PASI scores.

The different results in the parameters reported in the literature may be due to small numbers of subjects enrolled in the studies or to other patient factors, such as the type of psoriasis and the patient selection method. Factors relating to genetics, lifestyle, daily activities, and dietary issues may have also contributed to these dissimilar findings.

In the present study, CRP, MHR, NLR, and MLR were higher in patients with psoriasis than controls. All the parameters studied (CRP, MHR, NLR, PLR, and MLR) were positively correlated with PASI scores, which leads to the suggestion that these parameters may be used during the follow-up of psoriasis. Moreover, only MHR was positively correlated with CRP. Since CRP is a well-accepted marker of inflammation, it may be hypothesized that among the studied parameters, MHR tends to be more reliable in indicating the inflammatory status in psoriasis.

Apart from indicating ongoing inflammation, CRP, NLR, and PLR were shown to decrease after treatment with biologic agents for psoriasis, which is linked to the inhibition of systemic inflammation.^6^ On the basis of this data, it may be assumed that these parameters may determine not only the existing inflammation but may also provide a way to evaluate the effect of psoriasis treatment.

Since CRP, MHR, NLR, PLR, and MLR have been defined as prognostic markers of CVD, elevated values of these parameters may act as an alert in the detection and prevention of cardiovascular comorbidities.

The small sample size and the retrospective design of this study are limitations. The differing results that have been reported in the literature may be due to the subjective nature of the PASI evaluation and to the fact that the studies may have included different forms of psoriasis. Despite the small sample size of the study, the authors believe that the statistical analysis reveals important conclusions.

## Conclusion

Psoriasis is a systemic inflammatory disease associated with cardiovascular morbidities. In recent years, researchers have investigated laboratory biomarkers in psoriasis in addition to the clinical examination by PASI. Several cytokines and mediators, which are time- and cost-consuming to examine, have been determined in psoriasis. To the best of the authors’ knowledge, this is the first study evaluating CRP, MHR, NLR, PLR, and MLR all together in psoriasis. In the current study, CRP, MHR, NLR, and MLR were elevated and were correlated with PASI. It may be assumed that among the different ratios, MHR may be the most important since it is the only marker correlated with CRP, which may be due to the many functions of HDL-C, including antioxidative, anti-inflammatory, antiapoptotic, and antithrombotic functions.^31^ It is believed that MHR, NLR, and MLR, which can easily be accessed in daily practice, might be used to assess the severity of psoriasis in addition to the PASI. These parameters might also provide early detection of cardiovascular comorbidities in psoriasis.

## Financial support

None declared.

## Authors’ contribution

Ezgi Aktaş Karabay: Statistical analysis; approval of the final version of the manuscript; conception and planning of the study; composition of the manuscript; collection, analysis, and interpretation of data; participation in the design of the study; intellectual participation in the propaedeutic and/or therapeutic conduct of the studied cases; critical review of the literature.

Damla Demir: Approval of the final version of the manuscript; collection, analysis, and interpretation of data; participation in the design of the study.

Aslı Aksu Çerman: Approval of the final version of the manuscript; critical review of the manuscript.

## Conflicts of interest

None declared.
